# Supplementary material for: Do both timing and duration of screen use affect sleep patterns in adolescents?
Source: PLoS One. 2022 Oct 20;17(10):e0276226. doi: 10.1371/journal.pone.0276226 (PMC9584513; doi:10.1371/journal.pone.0276226)
Supplement: Attachment — Submitted filename: Review.docx [file pone.0276226.s002.docx]

Review: Do both timing and duration of screen use affect sleep patterns in adolescents

The authors investigate the involvement of screen usage in sleep disturbances in adolescents. A dose-response curve between levels of screen use on sleep deprivation and restriction was shown. The authors conclude that both screen use and timing are associated with adverse effects on sleep and daytime functioning. Overall, the study is interesting and valuable. However, the study design and interpretation does come with some clear limitations, as only subjective measurements and arbitrary cutoff points were used.

Major concerns

- Even though this is addressed in the discussion section of the manuscript, the biggest limitation of the study is that there are no objective measurements. As the authors address, it is difficult to estimate how well aware these adolescents are of their own screen usage. Even though the authors do state that there a modest association between self-report and logged screen time, can they at least speculate on the fact if these results might be over or under estimated?
- The cutoff points for various definitions are arbitrary. For example, excessive screen use is defined as >3 hours a day of screen use for social activities. If you use a different cutoff point, the results/interpretation might be completely different. I think the authors should elaborate on why certain thresholds were chosen, so that the reader for themselves can decide if they agree with these thresholds or not (for example “>3 hours defined as excessive was based upon a histogram distribution of all screen times, indicating that the top 5% was 3 hours or more”).
- The authors state that there is a dose-response relationship, but I have not seen a graph depicting this relationship. The authors should add this.

Abstract:

- “Sleep habits on school nights and weekends, symptoms of insomnia and daytime repercussions were recorded.” – please specify how

Implications et contribution:

- Please change to “Implication and contribution”

Introduction:

- I would suggest to rephrase “Sleep is regulated by the homeostatic and the circadian systems” to “Sleep is regulated by homeostatic sleep pressure and the circadian drive for sleep”, as homeostatic sleep pressure is not a system
- “the latter depends on external synchronizers or zeitgebers” – ref
- “For sleep to be restorative, the homeostatic and circadian systems need to be synchronized, and sleep time needs to be adequate” – this is not true, we actually don’t really know what constitutes restorative sleep. This may significantly differ from what is defined as restorative sleep as well, since objective and subjective measures differ. Please rephrase.

Materials and methods:

- “Daytime sleepiness was defined as a score >6 on a visual analogue scale” – is there a ref for this as well? Or is this just defined by the researchers?
- The materials and methods do not describe at all how the participants were separated into the different groups, while listed in the abstract as “(≥2 hours difference in sleep duration on school nights vs weekends)”. I am wondering what that cutoff is based upon and if that is a reasonable way of dividing people up.

Results

- Population and Sleep habits – please indicate median [IQR range] for demographic variables. That’s much more appropriate than the mean. Moreover, SD’s are not reported in the text.
- Also for table 1, I would recommend to report the median [IQR] since that is more appropriate and informative for population values
- “95.4% got up between 06:00 and 08:00 on school days, whereas 10% got up after 13:00 at the

Weekends” I wonder why the authors choose to name these specific values. To make a good comparison, would you not rather report when 95.4% got up on the weekend?

- “Before bedtime 11% read or listened to music, 30% did passive screen-based activities (ex. watching a film) 32% did active screen based activities (social networks, video games) and 27% did none of the above. A bedtime after midnight was much more common at the weekend (62% vs 14%).” – when you report that at this location, I also would like to know the duration of screen time usage, but in paragraph **screen use** such things are reported. I would therefore suggest to move this section.
- “Sleep restriction, defined as a reduction of >2 hours in sleep time on schooldays” – I think this should be defined in the materials and methods. Moreover, I don’t know how the authors came up with this cutoff point. Has that been previously reported in literature? Or is this a made-up cut-off point?
- “was intense screen use during the night” – but it has not been defined by the authors what is intense screen use. Also, for here, I wonder if this is based upon literature or not. If not, it should be stated clearly that these cut-off points have been designed by the authors.
- Table 3 & 4: could you add a column with the statistics?
- Since the authors claim there is a dose-response relationship, I would like to actually see a dose-response curve. The article would benefit significantly from a graph depicting the relationship between screen time and sleep and/or daytime functioning
- I’d like to see more results concerning the content. For me, this part is particularly interesting, since it can come with a lot of consequences. Can the authors show more of these data?

Discussion:

- “abolition of melatonin secretion which shifts the phase of the body clock” – well this significantly depends on the light intensity and spectral distribution of the screen. Please phrase more carefully
- “(our mean getting up time was 07:05)” – rephrase, since it was not you who got up at that time but the participants of the study.
- “and phase shifted circadian rhythms leading to delayed melatonin offset” – since you did not measure melatonin, you did not measure this. Therefore, you should rephrase. You’re not even sure that this is happening in this population.
- “The stimulating effects of light, modified melatonin secretion but also the mental stimulation of screen based activities may delay bedtimes” – again, this was not measured so please formulate more carefully
- “consequences. We found sleep onset insomnia in 16% of participants and a clear..” – why was this not described in the results section? You cannot report new results in the discussion. Please describe these results in the result section.
